# Physics-informed deep learning to forecast ${\widehat{{\varvec{M}}}}_{{\varvec{m}}{\varvec{a}}{\varvec{x}}}$ during hydraulic fracturing

**DOI:** 10.1038/s41598-023-40403-2

**Published:** 2023-08-12

**Authors:** Ziyan Li, David W. Eaton, Jörn Davidsen

**Affiliations:** 1 Department of Geoscience, University of Calgary, Calgary, AB T2N 1N4 Canada; 2 Department of Physics and Astronomy, University of Calgary, Calgary, AB T2N 1N4 Canada; 3 Hotchkiss Brain Institute, University of Calgary, Calgary, AB T2N 4N1 Canada

**Keywords:** Solid Earth sciences, Seismology

## Abstract

Short-term forecasting of estimated maximum magnitude (${\widehat{M}}_{max}$) is crucial to mitigate risks of induced seismicity during fluid stimulation. Most previous methods require real-time injection data, which are not always available. This study proposes two deep learning (DL) approaches, along with two data-partitioning methods, that rely solely on preceding patterns of seismicity. The first approach forecasts ${\widehat{M}}_{max}$ directly using DL; the second incorporates physical constraints by using DL to forecast seismicity rate, which is then used to estimate ${\widehat{M}}_{max}$. These approaches are tested using a hydraulic-fracture monitoring dataset from western Canada. We find that direct DL learns from previous seismicity patterns to provide an accurate forecast, albeit with a time lag that limits its practical utility. The physics-informed approach accurately forecasts changes in seismicity rate, but sometimes under- (or over-) estimates ${\widehat{M}}_{max}$. We propose that significant exceedance of ${\widehat{M}}_{max}$ may herald the onset of runaway fault rupture.

## Introduction

Hydraulic fracturing (HF), a fluid stimulation method to enhance permeability by producing fractures in low-permeability reservoir rocks^1^, typically produces microearthquakes (MEQs) with moment magnitude M_W_ < 0. However, HF can also induce moderate earthquakes (M_W_ > 4)^2–6^, which are associated with the activation of pre-existing faults^7^. Obtaining a probabilistic estimate of the largest expected event magnitude (${\widehat{M}}_{max}$) for a given HF operation is important for hazard assessment^8^ and could inform proactive real-time mitigation strategies for induced seismicity that are required in some advanced monitoring systems^9,10^.

Various approaches have been developed to estimate ${\widehat{M}}_{max}$ for fluid-induced seismicity. For example, the expected distribution of earthquake magnitudes can be expressed in terms of the net injected fluid volume (∆*V*) and the seismogenic index (∑), a proposed area-specific seismotectonic parameter that characterizes the expected seismic activity level in response to fluid injection^11^. This expression has been used to develop a probabilistic estimate for maximum magnitude^12^, which scales linearly with log_10_ ∆*V*. The same volumetric scaling relationship has been derived using a different theoretical approach, based on Griffith’s crack equilibrium criterion^13^. Here, the maximum magnitude estimate applies to the case of arrested rupture, a concept where the fault rupture zone is confined to a subsurface region in which pressure is perturbed by fluid injection. This concept has also been used to develop a geometrical constraint for maximum magnitude, based on the spatial distribution of MEQs^14^. In another formulation, the expected maximum seismic moment for an injection-induced earthquake is expressed as the product of the shear modulus of the medium and the net injected fluid volume^15^. With the exception of the geometrically constrained approach^14^, which requires MEQ hypocentre locations to be determined, all of these methods use net injected volume ∆*V* as a parameter for estimating ${\widehat{M}}_{max}$.

During HF operations, seismic observations can be used to identify operational MEQs^1^ as well as induced seismic events that occur on nearby faults^16–19^. Operational MEQs typically occur in clusters that extend away from the wellbore, usually perpendicular to the direction of minimum horizontal stress^20,21^. In some cases, a reactivated fault is characterized by delayed event occurrence relative to the start time of an injection stage, coupled with oblique orientation of seismicity clusters with respect to the principal stress directions^16–19^. Fault reactivation is often marked by an increase in seismicity rate, accompanied by a decrease in the Gutenberg-Richter *b*-value^22,23^. Although such changes in spatiotemporal pattern of seismicity may be subtle, their detection using deep learning (DL) methods could provide an avenue for improved short-term forecasting.

Li et al.^24^ developed a method for real-time operational forecasting of ${\widehat{M}}_{max}$ during stimulation. Their method estimates seismic efficiency ratio (SER), an empirically determined fraction of the maximum expected cumulative seismic moment based on injected volume^25^. The SER is calibrated during an initial time window of the injection program, and thereafter the maximum available seismic moment is assumed to be the difference between the projected seismic moment using the SER and the observed cumulative seismic moment. Like other methods discussed above, a disadvantage of this approach is that it requires access to injection volumetric data in real time, which may not be available to an independent observer.

To overcome this limitation, we propose two DL approaches for short-term forecasting of the expected maximum magnitude (${\widehat{M}}_{max})$ of induced seismic events during hydraulic fracturing. The first approach forecasts ${\widehat{M}}_{max}$ directly using DL. The second approach, which we refer to as physics-informed DL, uses DL to forecast the seismicity rate and then estimates ${\widehat{M}}_{max}$ using a formulation proposed by Van der Elst et al.^12^. This approach utilizes the maximum-likelihood value of ${\widehat{M}}_{max}$ and its associated probability distribution, assuming that earthquake magnitudes follow the Gutenberg-Richter (G-R) relationship. It relies on determination of the number of observed events (*N*_*c*_) within a certain time window that fall above the magnitude of completeness (*M*_*c*_) and the slope of the semilogarithmic magnitude-frequency distribution (*b*-value) from the G-R relationship. To enable the DL models to learn temporal patterns and trends in the seismicity data, we investigate two data partitioning methods to obtain sequential data samples for training and testing.

## Results

**Figure 1:**
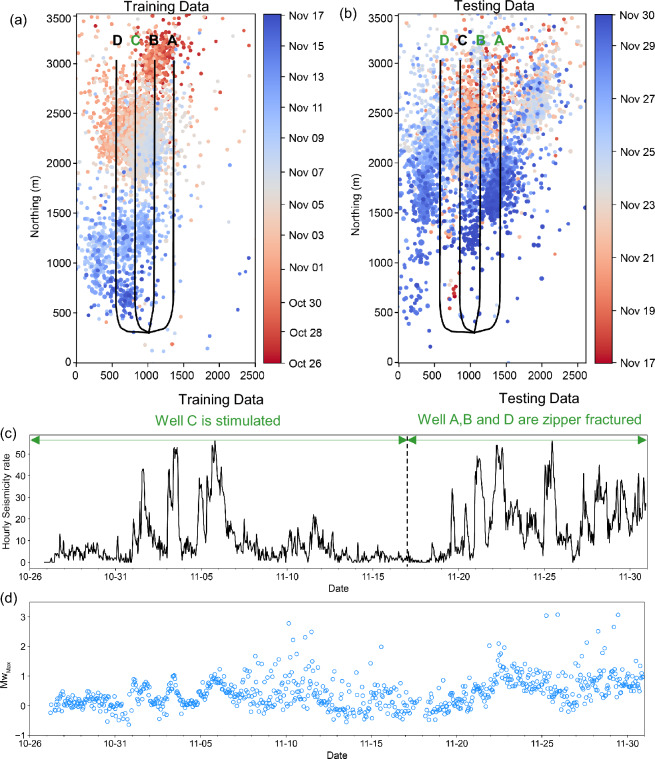
Induced seismicity dataset: (**a**) Seismic event epicenters for the training period, coloured by occurrence time. Four horizontal wells, drilled at ~ 3.4 km depth, are shown as black solid lines. (**b**) As in (**a**) but for the testing period. (**c**) Hourly seismicity rate. (**d**) Maximum moment magnitude during moving 1-h time windows.

To test our methods, we use observations of induced seismicity that occurred during HF treatment of four horizontal wells that were stimulated in 2016, over a period of four weeks^26^. Well C (Fig. 1) was stimulated first, in a series of stages from north to south, followed by wells A, B, and D, which were stimulated concurrently using a zipper-fracturing scheme^1^. For the training dataset, we use MEQs that occurred during HF of well C. Events that occurred during HF of wells A, B, and D provide the testing dataset for the DL models. The hourly seismicity rate varied between 0 and 60 events above the magnitude of completeness per hour, with maximum observed magnitude of M_W_ 3.1 (see “Methods”).

We apply both direct DL and physics-informed DL (PIDL) models to forecast the maximum likelihood value of ${\widehat{M}}_{max}$. The time-series input data is generated from the seismicity catalogue using two different data-partitioning methods. Method 1 scans the catalogue using a moving time window of fixed duration with a regular time step. The parameter of interest—i.e. the number of seismic events for PIDL model and the maximum magnitude for the direct DL model – is determined within each moving window frame. Method 2 uses a cumulative approach, where the window size is progressively increased by fixed duration at each step. We use a multi-layer perceptron network architecture for both DL models. This is a type of fully connected feed-forward artificial neural network with threshold activation ("Methods"). In the case of the direct DL, the output is the forecasted maximum magnitude. In the case of the PIDL model, to determine the maximum likelihood value of ${\widehat{M}}_{max}$ we make probabilistic short-term (a few hours) forecast using the formulation in^12^, as a function of the number of seismic events and the *b*-value. For both data partitioning methods, we consider two *b*-value scenarios, one where we use the directly estimated *b*-value of the current time window, and another one where we fix *b* = 1 to approximate a scenario involving fault activation^22^. Altogether, we evaluate six distinct approaches (Supplementary Materials, Table S1) that allow us to compare direct DL with physics-informed DL models, as well as the influence of data partitioning methods and the choice of *b*-value.

### Direct forecast

**Figure 2:**
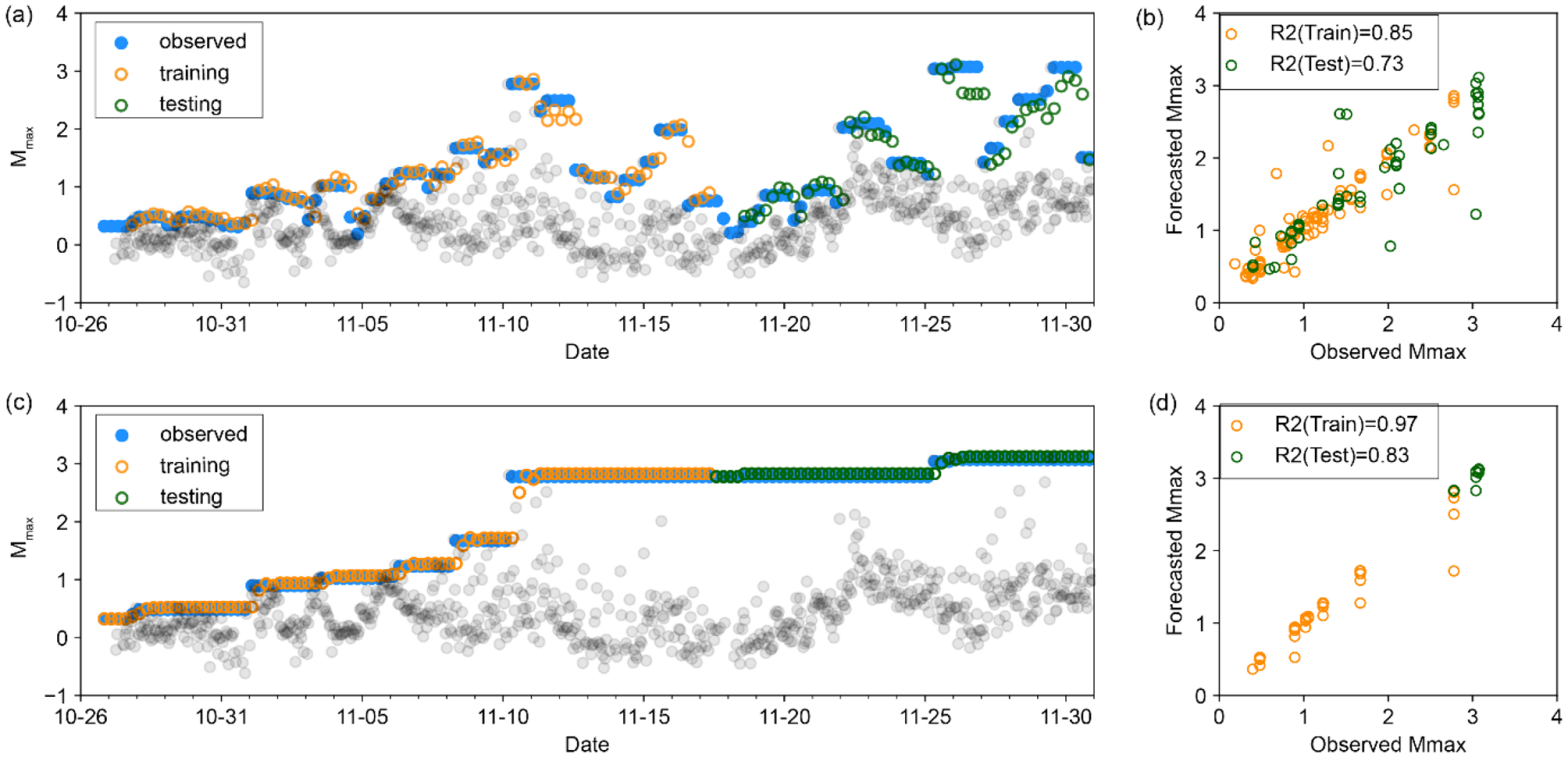
Direct DL models. (**a**) Forecasted ${\widehat{M}}_{max}$ for 24-h time windows using the fixed window method. Blue symbols represent the observed M_max_ in each time window. The orange and green symbols represent the forecasted ${\widehat{M}}_{max}$ for training and testing datasets, respectively. To show the seismicity with higher temporal resolution, the grey dots show the observed maximum moment magnitude within moving 1-h time windows. (**b**) Scatter plot comparing forecasted with observed ${\widehat{M}}_{max}$ using method 1. (**c**,**d**) As in (**a**,**b**), but using the cumulative data partition method.

The results of direct DL models to forecast ${\widehat{M}}_{max}$ are presented in Fig. 2. The first data-partitioning method estimates ${\widehat{M}}_{max}$ for a 24-h period, extending from 18 h prior to the current time to 6 h after the current time, while the second data-partitioning method uses a cumulative approach to forecast ${\widehat{M}}_{max}$. In the latter case, the calculated value of ${\widehat{M}}_{max}$ increases monotonically and covers a time window from the start of the analysis to 6 h ahead of the current time. Both data-partitioning methods exhibit higher *R*^2^ for training compared to the test set, which is normally expected. Overall, the R^2^ values are close to 1; however, when a jump occurs, there is a time lag between the observed and predicted values, reflecting the dominance of past observations included in the forecast time window. This time lag limits the practical utility of this direct DL approach for short-term operational forecasting of ${\widehat{M}}_{max}$.

### Physics-informed forecast

**Figure 3:**
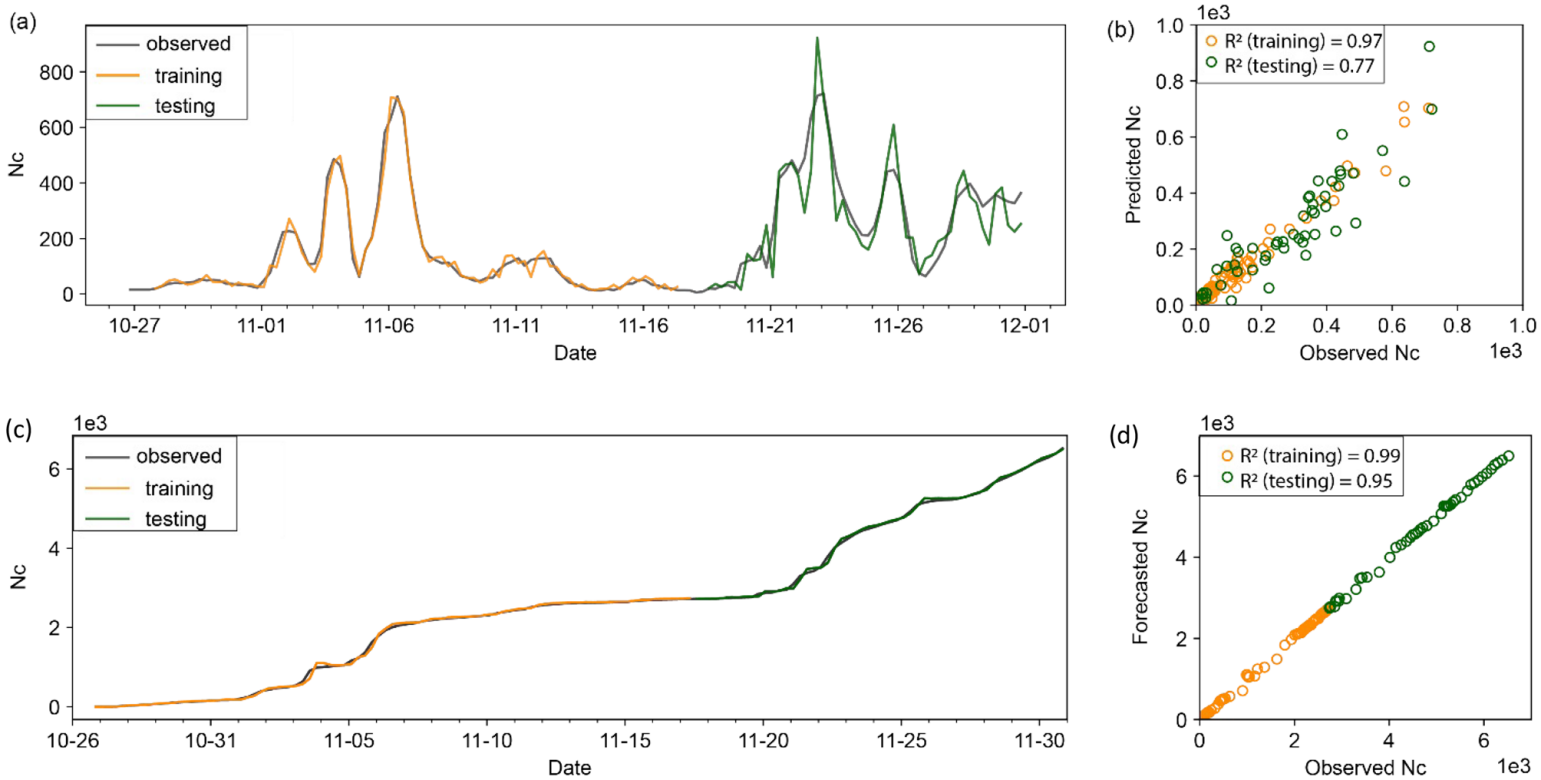
DL forecast for number of events ${\widehat{N}}_{c}$ above the magnitude of completeness. (**a**) Fixed time window method for the training and testing windows. (**b**) Scatter plot comparing the forecasted ${\widehat{N}}_{c}$ with measured measured *N*_*c*_. (**c**,**d**) As in (**a**,**b**) but using the cumulative data partition method.

Instead of directly forecasting ${\widehat{M}}_{max}$, the physics-informed approach uses DL to forecast the seismicity rate (represented here by the number of events above the magnitude of completeness within the current time window, *N*_c_), from which ${\widehat{M}}_{max}$ is estimated. Figure 3 shows the time series for the observed value of *N*_c_ and the forecasted value of ${\widehat{N}}_{c}$, for both data-partitioning methods. In the case of the fixed-window method, *N*_c_ fluctuates, as expected for HF stimulations that vary in location and intensity, whereas for the cumulative method, *N*_*c*_ and ${\widehat{N}}_{c}$ both increase monotonically. The higher *R*^2^ value for method 2 reflects the preponderance of prior data within the cumulative time window.

**Figure 4:**
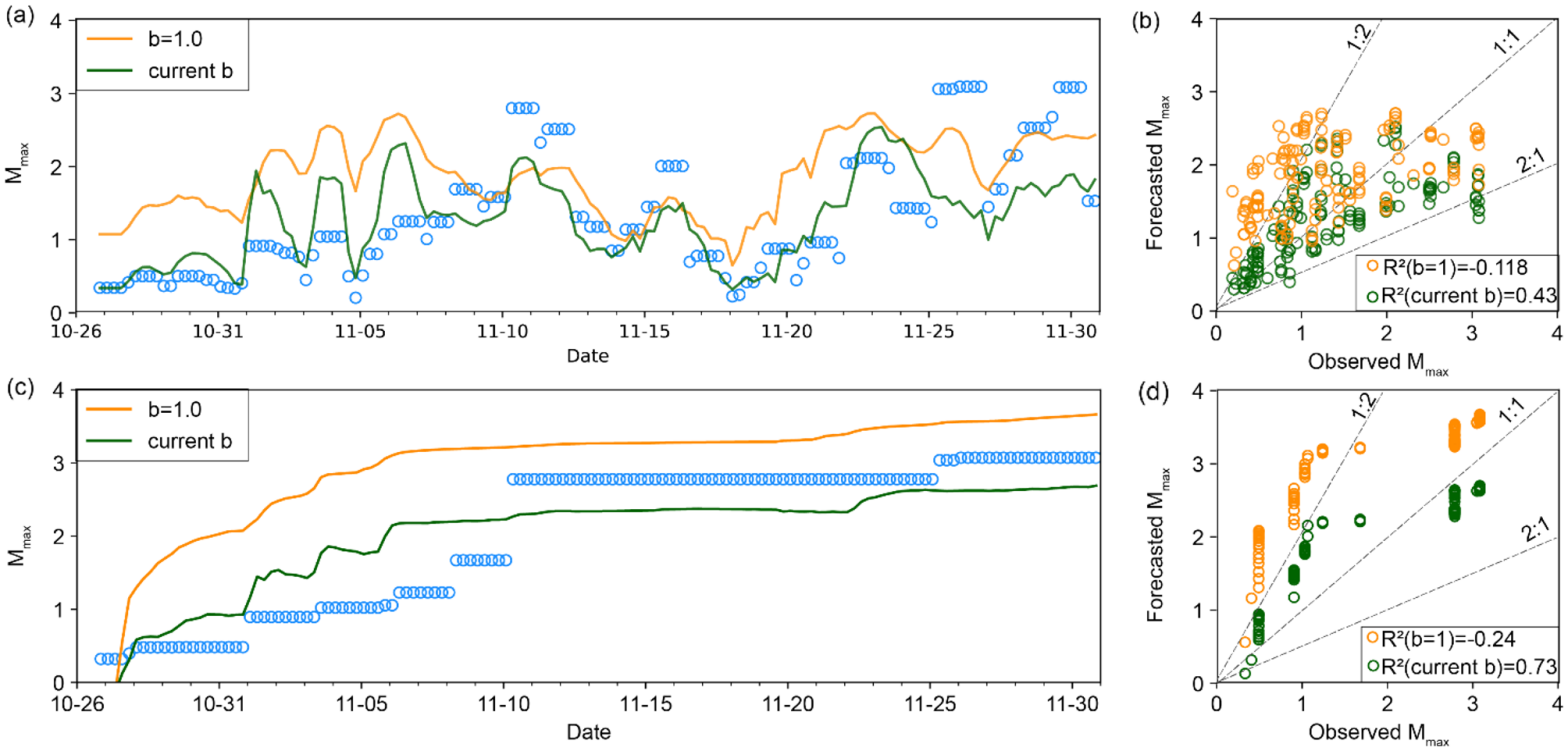
Physics-informed DL (PIDL) models to forecast ${\widehat{M}}_{max}$. (**a**) The PIDL model to forecast ${\widehat{M}}_{max}$ for 24-h time windows using fixed (orange) and floating (green) *b*-values. Blue symbols represent the observed *M*_*max*_ in each time window. (**b**) Scatter plots of forecasted ${\widehat{M}}_{max}$ vs. measured *M*_*max*_ for the fixed window approach. (**c**,**d**) As in (**a**,**b**) but using the cumulative data partition method.

Using ${\widehat{N}}_{c}$, we can calculate ${\widehat{M}}_{max}$ based on the GR relation for different choices of *b-*value ("Materials and methods"). Figure 4 shows two scenarios, one that uses a maximum-likelihood floating estimate of the *b*-value^27–29^ for the current time window, and another with a fixed value of *b* = 1 that is broadly representative of fault activation^22^. To forecast ${\widehat{M}}_{max}$, the use of a fixed *b*-value allows for faster response with a margin of safety, since accurate determination of *b* requires a relatively large sample size (> 1000 MEQs)^30^. The fixed-window method with a floating estimate of *b* appears to track temporal fluctuations for small seismic magnitudes (M_W_ < 2), but it fails to forecast larger events (Fig. 4a). This can be ameliorated by fixing *b* to unity, which leads to a forecast that approximates the upper limit for most seismic events but still fails to provide a forecast envelope for the largest observed events. For the cumulative data partition method, ${\widehat{M}}_{max}$ increases monotonically over time (Fig. 4c), as expected. In all cases the forecast has a low R^2^ value (Fig. 4b,d), indicating that for this approach the calculated value is not suitable for a direct forecast, although it could provide a forecast of the envelope of ${\widehat{M}}_{max}$.

**Figure 5:**
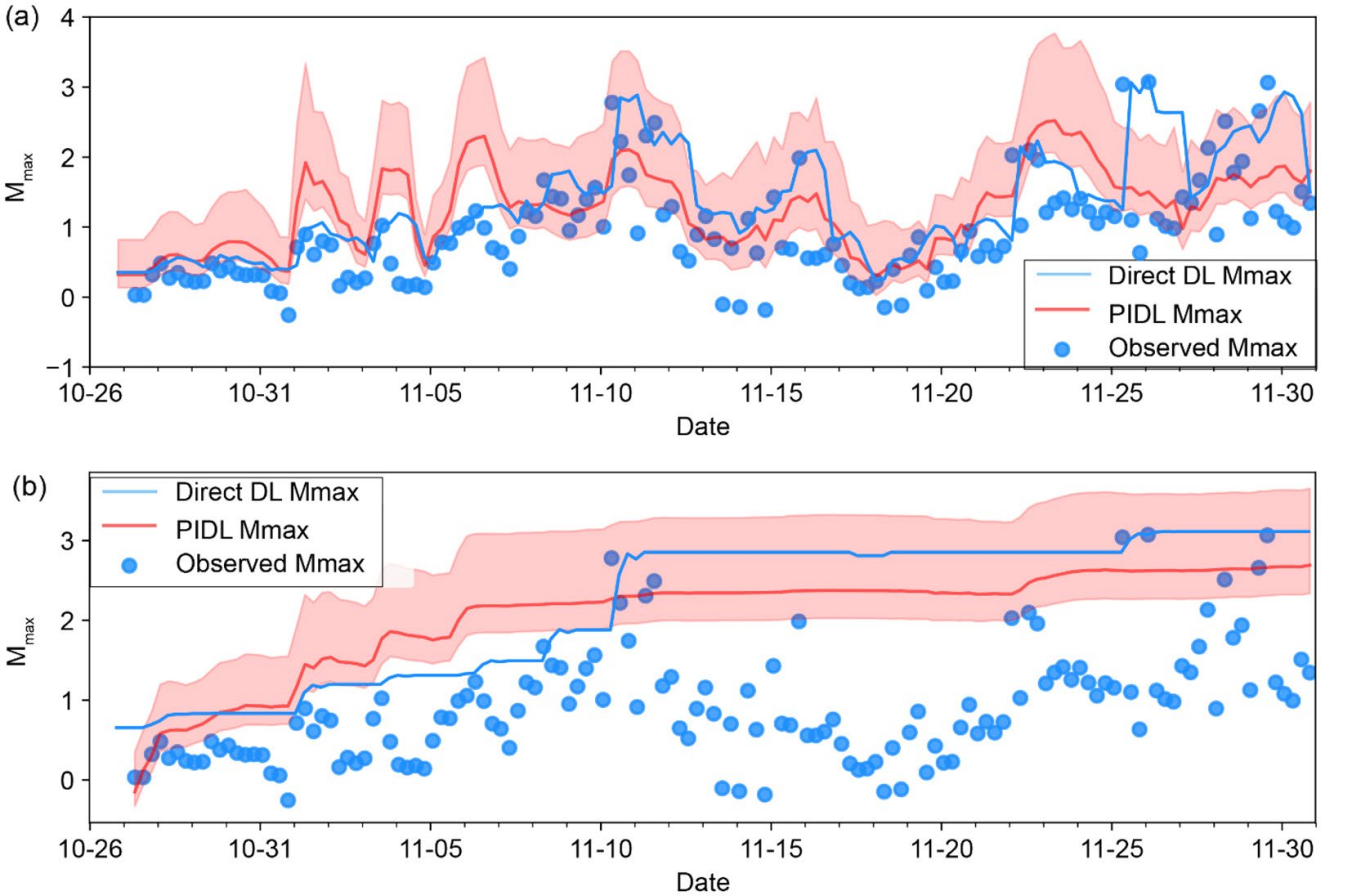
Comparison of direct and physics-informed deep learning (PIDL) models for forecasting ${\widehat{M}}_{max}$. (**a**). Fixed-window calculations, where the blue symbols show the maximum magnitude in 6-h time windows and the shaded region shows a forecast envelope based on a 95% confidence region for the PIDL curve, assuming time-varying *b*-value. (**b**) As in (**a**) for the cumulative approach.

Figure 5 shows DL and PIDL results, highlighting the 95% confidence region for the PIDL calculation based on the GR magnitude distribution^12^. As noted previously, the DL method provides a closer fit to the observed magnitude distribution, but a time lag limits the utility of this approach for forecasting purposes. In the case of this field experiment^26^, a TLP was in effect at the time of the HF program^31^, with a *M*_*L*_ 2.0 yellow-light threshold requiring reduced operations and a *M*_*L*_ 4.0 red-light threshold requiring operational suspension. Under this TLP, the yellow-light condition was triggered on 2016/11/10 and on 2016/11/25. The red-light threshold was not exceeded.

## Discussion

Traffic-light protocols (TLPs) are a reactive-control approach used to mitigate risks of induced seismicity based on discrete response thresholds that invoke a specific action, such as modifying (or suspending) HF stimulation upon the occurrence of events that exceed a specific magnitude^1^. Using this framework, approaches to mitigate risk, as opposed to hazard, have recently been introduced^32^. Although TLPs have been implemented in many jurisdictions to manage induced seismicity risks associated with hydraulic fracturing^6,33^ or enhanced geothermal systems^34^, their underlying assumptions have been called into question; for example, most TLPs are based on a tacit assumption that anomalous larger events are preceded by weaker precursory seismicity, or that curtailment of fluid injection will necessarily lead to an immediate reduction in the level of seismicity^35,36^. Occurrence of HF-induced seismicity in the presence of a TLP show that these assumptions are not universally applicable^37^. Adaptive (or advanced) TLPs have been proposed^9,10,24,38^, but these methods require real-time access to stimulation data, such as the injection rate. Since this type of data is not always available in real time to an independent observer, we focus here on a purely data-driven approach.

The fixed-window PIDL calculations (Fig. 5a) exhibit fluctuating levels of ${\widehat{M}}_{max}$ that we propose could serve as the basis for a type of adaptive TLP system. For example, based on the 95% confidence region using the fixed-window PIDL approach, the yellow-light threshold was exceeded during the training period on 2016/11/01, and it was exceeded during the testing period on 2016/11/22. Had this PIDL-based criterion been used to trigger TLP responses, it would have provided a forecast with several days of advance notice to apply operational reduction during the testing period. Although the floating *b*-value PIDL method is shown, essentially the same advance notification would apply using a fixed value of *b* = 1 (Fig. S4), despite its more conservative nature. The use of a training data set restricted to a single well further shows that this advance notification successfully extends to other wells and different injection protocols in the same geological setting suggesting that the fixed-window PIDL approach has a degree of transferability.

The phenomenon of runaway rupture, wherein the slip region of an induced earthquake outgrows the fault area perturbed during stimulation^13^, can lead to exceedance of forecast magnitudes^7^. In this scenario, the maximum slip surface area is limited by the physical fault dimensions rather than stimulation parameters^12^. For example, the 2017 MW 5.5 Pohang earthquake in Korea has been cited as an example of runaway rupture^39^. As illustrated in Fig. 5, four events occurred during this HF program that exceed the magnitude range of the 95% confidence region based on the fixed-window PIDL approach. As a proof-of-principle, we interpret such exceedance to herald the possible onset of runaway fault rupture; accordingly, an adaptive TLP developed using this PIDL approach could incorporate a red-light threshold using this criterion rather than a specific fixed magnitude level. Further testing is needed to establish the robustness of this observation.

In summary, our findings provide a proof-of-principle that a fixed-window PIDL approach could serve as a basis for an adaptive TLP system. While many studies have explored methods to forecast induced seismicity associated with industrial activity, including hydromechanical models that combine fluid pressurization and rate-and-state friction^40^ or related mechanisms^41,42^, models to predict maximum seismic magnitude using injection data^12,15,24,25^ and machine learning models to forecast induced seismicity rates using highly related features^43^, these models require access to injection data and/or geomechanical parameters, e.g. poroelastic stress, stress rate and rate-state friction parameters, which are typically not available at all or at least not in real-time. In contrast, our PIDL approach to forecast ${\widehat{M}}_{max}$, while requiring a training period, is exclusively based on the observed seismic catalog and allows real-time forecasting. Using a fixed *b*-value further eliminates the potential pitfall of large uncertainties arising from estimating it over small time windows adding to the robustness of the PIDL approach.

## Methods

### Study area

The HF treatment we analyze here is the Tony Creek Dual Microseismic Experiment (ToC2ME) dataset^26^, which was acquired by the University of Calgary in 2016. This HF simulation program is located within the Duvernay shale play in western Canada, within an area noted for susceptibility to HF-induced seismicity^2,3,6^. The ToC2ME acquisition systems included a 68-station shallow borehole array, six broadband seismometers, and one strong-motion accelerometer^26^. A resulting seismicity catalog obtained using an automated method^44^ contains > 10,000 events, with a maximum magnitude of 3.1 MW. Overall, the observed seismicity is characterized by b >  > 1, as expected for operational MEQs^1^; however, individual event clusters associated with fault activation show a marked drop in b-value^45^. Based on the b-value stability method^28,29^ and the maximum likelihood method^27^, we determine Mc using the first 1000 MEQs in the catalogue finding Mc = – 0.15 (Fig. S1). Since the sensors used in the study are fixed and the event depths remain approximately the same throughout the HF program^46^, we assume that Mc is fixed at this value (– 0.15) for the duration of the experiment.

### DL model framework

We use multi-layer perceptron (MLP) networks^47^, which are a fully connected class of feedforward artificial neural network composed of multiple layers of perceptron with threshold activation. MLP networks learn a function that maps a sequence of input observations to an output observation.

Each MLP network consists of one input layer, two hidden layers, and one output layer. The input layer receives data and passes it to the first layer. The hidden layers perform mathematical computations on inputs and return a forecasted value as the output layer. Each neuron is fully connected to all the neurons in the preceding layer and those in its next layer. The neuron combines input with weights and outputs a value from the activation function of the sum of input-weight products. The output ${\widehat{h}}_{out}$ can be generally expressed as

1 $${\widehat{h}}_{out}=f\left(\sum_{i=1}^{n}{w}_{i}{x}_{i}+b\right),$$

where $w$ is the weight of input *x*, *b* is the bias, *n* is the number of inputs, $f$ is the activation function used to standardize the output coming out of the neuron.

We apply the rectified linear units (ReLU) activation function for the two hidden layers to perform the forecast, which is linear for all positive values and zero for all negative values. Mathematically, it is defined as

2 $$f\left(x\right)=\mathrm{max}\left(0,x\right).$$

The algorithm consists of feedforward and backpropagation phases. In the feedforward phase, inputs are combined with the initial random weights in a weighted sum and subjected to the activation function. The outputs from neurons are then used as inputs to the next layer. Each layer feeds the next one with the result of their computation, which goes through the hidden layers to the output layer. The error of the forecasted output is stored. We apply a Mean Squared Error (MSE) loss function in 1st MLPs to calculate the output,

3 $${E}_{Nc}\left({y}_{t},\widehat{{y}_{t}} \right)=\frac{1}{N}\sum_{N}{({y}_{t}-\widehat{{y}_{t}})}^{2}.$$

In the backpropagation phase, the errors evaluate the derivatives of the loss function with respect to the weights ${\nabla }_{loss}$. The gradient then updates the weights with respect to the loss function,

4 $${w}_{t} = {w}_{t-1}-\alpha {\nabla }_{loss},$$

where ${w}_{t}$ is the gradient at current iteration, ${w}_{t-1}$ is the gradient at the previous iteration, and α represents the learning rate,

5 $${\nabla }_{loss}=\frac{dE\left({y}_{t},\widehat{{y}_{t}} \right)}{dw\left(t\right)}.$$

In each iteration, the gradient is computed across all input and output pairs after the weighted sums are forwarded through all layers until the output is estimated. As an example, the specific inputs and outputs for the different DL models are given in Table S1 and Figs. S2 and S3. The weights of the first hidden layer are updated with the value of the gradient, i.e. we use the efficient Adam stochastic gradient descent^47^ optimized using MSE. This process continues until the gradient for each input–output pair converges, meaning the newly computed gradient has not changed more than a specified convergence threshold compared to the previous iteration.

### PIDL models

For the PIDL models, rather than forecasting ${\widehat{M}}_{max}$ directly, we estimate the number of events (${\widehat{N}}_{c})$ above the magnitude of completeness (*M*_*c*_) for a time period that extends into the future ahead of the current time. The value of ${\widehat{N}}_{c}$ is then used to estimate the corresponding maximum-likelihood value of ${\widehat{M}}_{max}$ using a formula developed in^12^,

6 $${\widehat{M}}_{max}= {M}_{c}+\frac{1}{b}{log}_{10}{\widehat{N}}_{c}.$$

*M*_*c*_ is treated as a constant (and estimated from the data), whereas two different approaches are used for the *b* value in this expression. In the first approach a current estimate of *b* is obtained from the seismicity catalogue. In the second approach, the *b* value is set to 1 to consider the possibility of an abrupt reduction in *b* value due to fault activation. In practice, such a reduction in *b* value cannot be detected immediately due to a time lag imposed by the requirement for the number of observations to estimate *b* in a robust manner^30^. This approach also provides an estimate for the maximum magnitude bounds for a specific confidence level *q*, expressed as

7 $${M}_{q}={M}_{max}-\frac{1}{b}{\mathrm{log}}_{10}\left(-\mathrm{ln}q\right).$$

### Supplementary Information

Supplementary Information.

## Data Availability

Data and codes supporting the findings of this manuscript are available from the corresponding author upon request. The seismic catalog for this study (Catalog_Rodriguez-Pradilla2019_PhDThesis.csv) can be found at: https://github.com/ToC2ME/ToC2ME/tree/master/Rodriguez-Pradilla.
