# Supplementary material for: Physics-informed deep learning to forecast M^max during hydraulic fracturing
Source: Sci Rep. 2023 Aug 12;13:13133. doi: 10.1038/s41598-023-40403-2 (PMC10423224; doi:10.1038/s41598-023-40403-2)
Supplement: Supplementary file 1 — Supplementary Information. [file 41598_2023_40403_MOESM1_ESM.pdf]

Supplementary Materials for

Physics-informed deep learning to forecast $\widehat{M}_{max}$ during hydraulic fracturing

Ziyan Li *et al.*

*Corresponding author. Email: eatond@ucalgary.ca

This PDF file includes:

Table S1
Figs. S1 to S5

Table S1. Six deep learning scenarios tested in this study.

case No.	Methods	Input	output	Data partition methods	b-value
1	direct DL	Mmax	Mmax	method 1	-
2	direct DL	Mmax	Mmax	method 2	-
3	PIDL	Nc	Mmax	method 1	Fixed at 1
4	PIDL	Nc	Mmax	method 1	current b
5	PIDL	Nc	Mmax	method 2	Fixed 1
6	PIDL	Nc	Mmax	method 2	current b

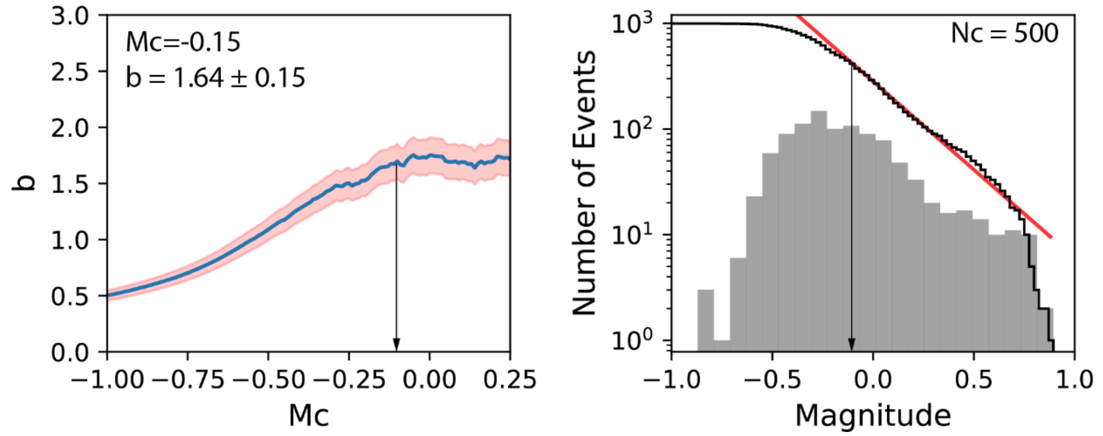

Fig. S1. Frequency-magnitude characteristics. (a) Plot of *b*-value vs. M_c calculated using the first 1000 MEQs. The maximum-likelihood *b*-value **(***b* = 1.64) corresponding to M_c= –0.15 is representative of the entire dataset, as expected for operationally induced MEQs. (b) Cumulative (black curve) and non-cumulative (shaded area) distribution of event magnitudes, showing best-fitting Gutenberg-Richter relationship (red line). The indicated uncertainty of the *b*-value (0.15) represents the 95% confidence limit.

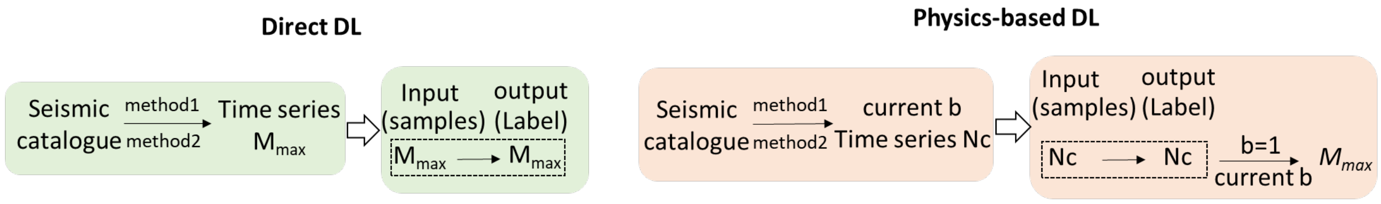

Input (samples)	Output(labels)
[Mw(t0),Mw(t1),Mw(t2),Mw(t3)]	Mw(t4)
[Mw(t1),Mw(t2),Mw(t3),Mw(t4)]	Mw(t5)
[Mw(t2),Mw(t3),Mw(t4),Mw(t5)]	Mw(t6)
[Mw(t3),Mw(t4),Mw(t5),Mw(t6)]	Mw(t7)
[Mw(ti-3),Mw(ti-2),Mw(ti-1),Mw(ti)]	Mw(ti+1)

Training Data
Testing Data

Input (samples)	Output(labels)
[Nc(t0),Nc(t1),Nc(t2),Nc(t3)]	Nc(t4)
[Nc(t1),Nc(t2),Nc(t3),Nc(t4)]	Nc(t5)
[Nc(t2),Nc(t3),Nc(t4),Nc(t5)]	Nc(t6)
[Nc(t3),Nc(t4),Nc(t5),Nc(t6)]	Nc(t7)
[Nc(ti-3),Nc(ti-2),Nc(ti-1),Nc(ti)]	Nc(ti+1)

Training Data
Testing Data

Fig. S2. Step by step description of direct DL and PIDL methods. The parameter of interest – i.e., the number of seismic events for the physics-informed DL (PIDL) model and the maximum magnitude for the direct DL model – is determined within each moving window frame. Both direct DL and PIDL models to map the sequence of input observations to output observations. In the case of the direct DL, the output is the forecasted maximum magnitude, whereas for the PIDL it is the forecasted number of seismic events in a given time window, above the magnitude of completeness. Direct DL and PIDL inputs and output data sequence are shown in the corresponding table.

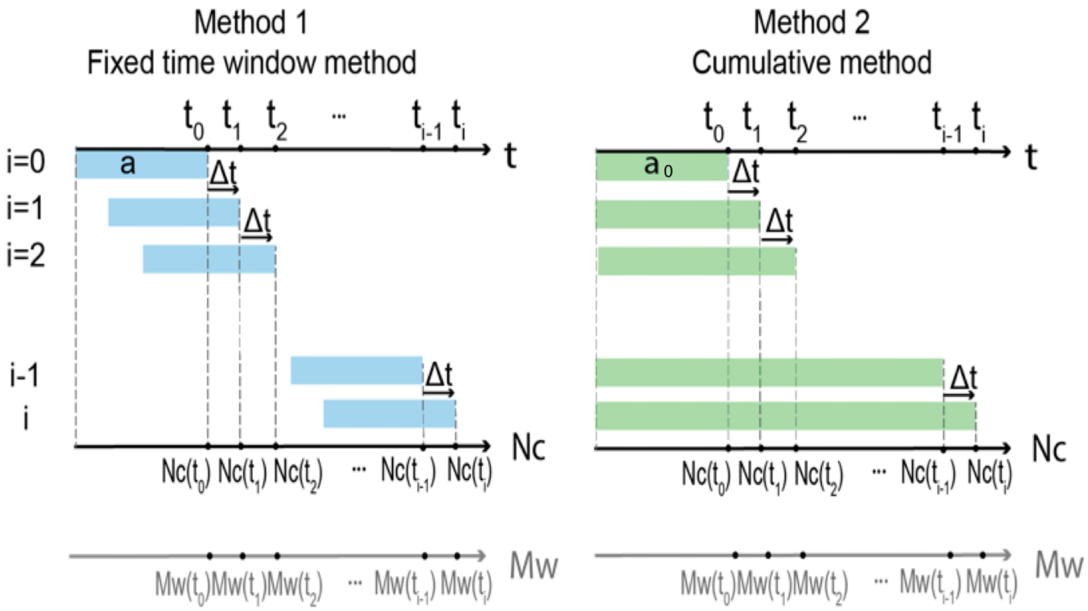

Fig. S3. Data partition methods. Method 1 is a fixed-window method, where the seismicity catalog is scanned using moving time windows with fixed window size. Method 2 uses a cumulative approach, where the temporal length of the window increases by a specific fixed time interval per step.

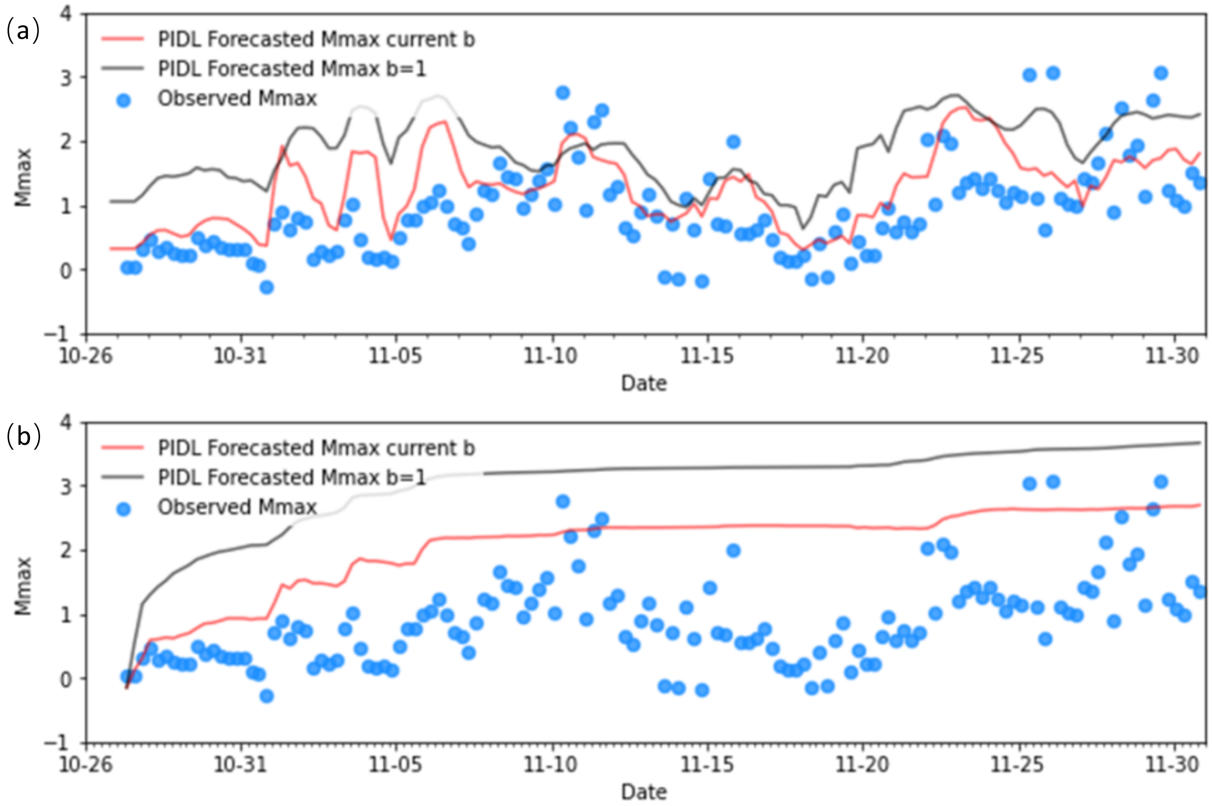

Fig. S4. Impact of *b*-value selection on the performance of PIDL models for forecasting $\widehat{M}_{max}$. (a). Fixed-window calculations, where the blue symbols show the maximum magnitude in 6-hour time windows and the shaded region shows a forecast by PIDL model using time-varying *b*-value (see Fig. S5 for the actual estimates) and b=1. (b) As in (a) for the cumulative approach.

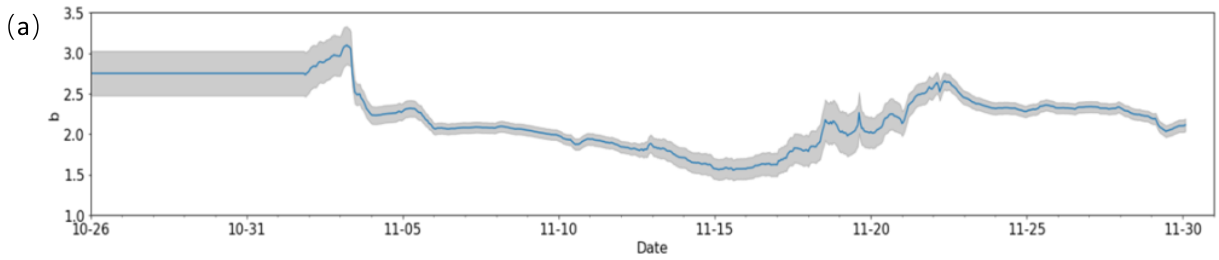

Fig. S5. Plot of *b* value for partition methods. The time dependent *b* value is calculated based on method 1 (a) and method 2 (b). Shaded region shows 95% confidence interval.
